# Edge effects and mating patterns in a bumblebee-pollinated plant

**DOI:** 10.1093/aobpla/plaa033

**Published:** 2020-07-03

**Authors:** Dorothy A Christopher, Randall J Mitchell, Dorset W Trapnell, Patrick A Smallwood, Wendy R Semski, Jeffrey D Karron

**Affiliations:** 1 Department of Biological Sciences, University of Wisconsin–Milwaukee, Milwaukee, WI, USA; 2 Department of Biology, University of Akron, Akron, OH, USA; 3 Department of Plant Biology, University of Georgia, Athens, GA, USA

**Keywords:** Edge effects, gene dispersal, mate diversity, *Mimulus*, monkeyflower, paternity, pollination, seed set, siring success, spatial location

## Abstract

Researchers have long assumed that plant spatial location influences plant reproductive success and pollinator foraging behaviour. For example, many flowering plant populations have small, linear or irregular shapes that increase the proportion of plants on the edge, which may reduce mating opportunities through both male and female function. Additionally, plants that rely on pollinators may be particularly vulnerable to edge effects if those pollinators exhibit restricted foraging and pollen carryover is limited. To explore the effects of spatial location (edge vs. interior) on siring success, seed production, pollinator foraging patterns and pollen-mediated gene dispersal, we established a square experimental array of 49 *Mimulus ringens* (monkeyflower) plants. We observed foraging patterns of pollinating bumblebees and used paternity analysis to quantify male and female reproductive success and mate diversity for plants on the edge versus interior. We found no significant differences between edge and interior plants in the number of seeds sired, mothered or the number of sires per fruit. However, we found strong differences in pollinator behaviour based on plant location, including 15 % lower per flower visitation rates and substantially longer interplant moves for edge plants. This translated into 40 % greater pollen-mediated gene dispersal for edge than for interior plants. Overall, our results suggest that edge effects are not as strong as is commonly assumed, and that different plant reproduction parameters respond to spatial location independently.

## Teaser

Many flowering plant populations have irregular shapes that increase the proportion of plants on the edge. This reduces the number of neighbours a plant has, which may reduce mating opportunities and lead to decreased fitness. Plants that rely on pollinators may be particularly vulnerable to edge effects. Christopher *et al.* examined whether fitness via male and female function as well as pollinator foraging patterns differed between plants on the edge or the interior of an experimental population. They found no differences between fitness components. However, pollinator visitation rates were significantly lower for edge plants. This suggests that plant reproduction parameters respond independently to spatial location.

## Introduction

Many flowering plant populations are small in size, or have a linear or irregular shape, characteristics that increase the proportion of individuals on the population’s edge (Handel 1983). These ‘edge’ plants have fewer neighbours than ‘interior’ plants, potentially altering pollinator foraging behaviour, and reducing mating opportunities and mate diversity through both male and female function (Aldrich and Hamrick 1998; Cresswell 2000; Ison and Wagenius 2014). Reduced mating opportunities limit the options for mate choice, and reduced mate diversity may influence the likelihood of successful offspring establishment in spatially heterogeneous environments. Both are thought to be important components of reproductive success (Karron and Marshall 1993; Pannell and Labouche 2013; Krauss *et al.* 2017).

**Figure 1.**
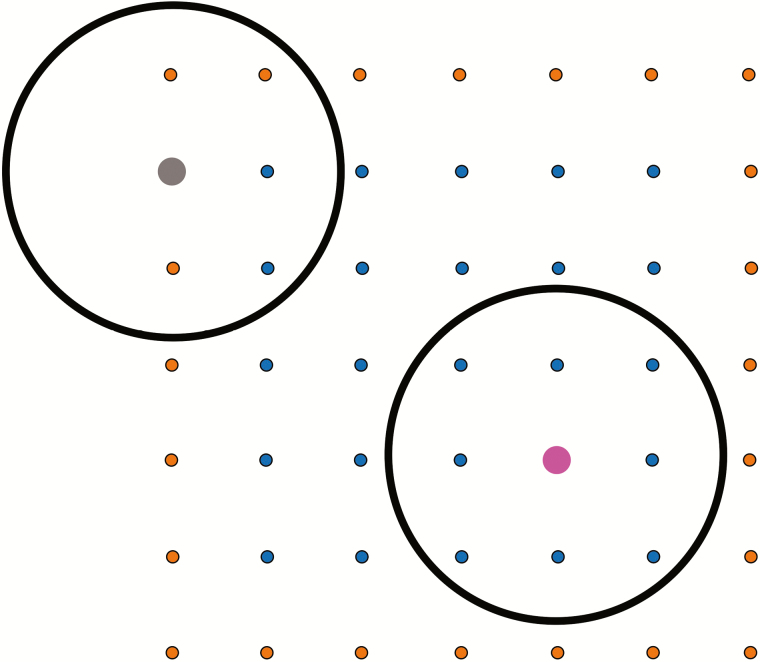
Schematic of the experimental array, a 7 x 7 square grid of plants spaced 0.8 m apart. Edge plants are shown in orange; interior plants in blue. A focal plant on an edge of the population (shown in grey) has five adjacent neighbours. A focal plant in the interior of the population (shown in pink) is surrounded by eight near neighbours.

Plants that rely on pollinators exhibiting area-restricted foraging (such as bumblebees; Levin and Kerster 1969a, b; Karron *et al.* 1995) may be especially susceptible to ‘edge’ versus ‘interior’ position effects (Cresswell 2000, but see Hodges and Miller 1981). In an evenly spaced population resembling a square grid, edge plants have only 62 % as many near neighbours as those in the interior (five near neighbours rather than eight; Fig. 1). When pollen carryover is limited so that most pollen dispersed from a focal plant is deposited on stigmas of the next plant the bee visits (Thomson and Plowright 1980; Holmquist *et al.* 2012), the number of potential mates would be reduced. However, if pollen carryover is more extensive, the number of plants immediately adjacent to ‘edge’ plants becomes less important because a sizeable fraction of pollen from ‘edge’ plants may be dispersed to other more distant recipients (or is received by ‘edge’ plants from more distant pollen donors; see Levin 1995). Although previous work has quantified seed production (e.g. Kunin 1997; Burgess *et al.* 2006; Ison and Wagenius 2014; Gargano *et al.* 2017) and mate diversity (Barriball *et al.* 2014) for edge versus interior plants, the effects of spatial position on siring success and the extent of pollen-mediated gene dispersal have not previously been explored.

Here we use parentage analysis to quantify male and female reproductive success and mate diversity for edge and interior plants in an experimental population of a bumblebee-pollinated hermaphroditic plant. To assess the role of pollen carryover, we also contrast pollinator flight movements and gene dispersal following visits to edge and interior plants. We address the following questions: (i) Do edge and interior plants differ in male and female reproductive success? (ii) Do edge and interior plants differ in mate number (sires per fruit)? (iii) Do mean pollinator flight distances differ following departures from edge and interior plants? (iv) Do edge and interior plants differ in patterns of pollen-mediated gene dispersal?

## Methods

### Study system

*Mimulus ringens* (Phrymaceae) is a diploid, self-compatible, wetland perennial native to central and eastern North America. It produces zygomorphic purple flowers that last a single morning and are pollinated primarily by bumblebees. At our field site the most common pollinators are *Bombus vagans* and *B. impatiens*, with *B. fervidus*, *B. griseocollis*, *B. pennsylvanicus* visiting less frequently (Mitchell *et al.* 2004). Flowers generally receive 1–3 bumblebee visits before stigmas close in the late morning (Mitchell *et al.* 2005; Karron *et al.* 2006). *Mimulus ringens* populations are typically composed of 50–2000 individuals and have shapes ranging from linear and only 1–5 plants wide (streamside, edge of a steep vertical gradient, or narrow depressions and channels) to nearly circular and >40 plants wide (marshes and wet meadows; pers. obs.).

### Experimental design

We constructed an experimental array of 49 *M. ringens* genets in a common garden at the University of Wisconsin–Milwaukee Field Station (Saukville, WI, USA; 43.387335°N, 88.022870°W). The array is surrounded by a restored prairie with many bumblebee-pollinated species. No natural populations of *M. ringens* occur within 12 km of our study site. Experimental plants were grown to flowering in 20-cm pots. These plants were grown from seed collected from a single natural population at the Panzner Wetland Wildlife Reserve (Akron, OH, USA; (41.068524°N, 81.612118°W). Plants in this population have 1–2 flowered displays and produce 6–8 flowers over a season.

We conducted the experiment on four fair weather days between 25 July 2017 and 31 July 2017. We randomly assigned positions to genets in the array and re-randomized the array each subsequent day to minimize confounding of genet and location effects. On each day, we trimmed plants to a single flower before anthers dehisced and before pollinators began visiting flowers at sunrise (0530 h).

### Pollinator observations

We observed pollinator visitation patterns each day between 0530 and 1000 h; this is the window of time in which pollinator visits provide effective pollination. Flowers usually receive 1–3 visits during this 4.5-h period (Karron *et al.* 2006). By 1000 h all stigmas were closed.

Each day of the study three observers recorded all pollinator visits during 15-min intervals spaced throughout the morning, resulting in four 15-min observation periods per day. When a bee entered the array, one of the observers followed and recorded the bee species and the entire visitation sequence. The observer recorded the unique identification and location of the first plant the bee visited and the time of that visit, and then recorded all subsequent plants visited in the order in which the bee visited. When the bee departed the array, the observer recorded the time of departure. From the plant location information, we could then calculate how far the bee travelled in metres. While that observer was occupied, the other observers scanned for and followed any other bees in the array. In almost all cases there were fewer than two simultaneously foraging bees, so full visitation sequences were scored. In the rare instances when more bees were present at once, the observers recorded which plants were visited but were unable to record simultaneously the order of plants visited by each individual bee. We are confident that we recorded all visits even under those circumstances.

### Quantifying female reproductive success

We tagged flowers at 1300 h after stigmas had closed and effective pollination for the day was completed. On all 4 days, every flower produced a fruit, for a total of 196 fruits. We collected the fruits on 28 August 2017 to 30 August 2017. Three of the 196 fruits suffered damage by seed predators and could not be used for quantifying seed number or siring success.

We used image analysis to count the thousands of minute seeds in each fruit. To do this we scraped seeds from the fruit and placed them in a transparent zippered plastic bag to facilitate handling. We then scanned the bag with the seeds at 600 DPI with a flatbed scanner (HP 9000T), and counted the seeds using ImageJ software version 1.5.2r (Schneider *et al.* 2012). Final seed counts represent means of five scans of the seeds of each fruit (scan counts match hand counts closely, *r* = 0.97, *N* = 20).

### Quantifying male reproductive success and mate number per fruit

To assess paternity, we genotyped 5 seedlings per fruit (965 seedlings total) with eight microsatellite loci following the methods of Nunziata *et al.* (2012). Genotypes of all maternal plants were known (Christopher *et al.* 2019). The multilocus exclusion probability given known maternal genotypes was 0.98. There was 1 % missing data in the final data set.

We performed paternity analysis to identify the most likely father of each seedling using the maximum likelihood procedure in Cervus v3.0 (Kalinowski *et al.* 2007). We retained the default 2 % genotyping error rate. The program successfully assigned paternity to a single father for each of 842 seedlings (87 % of the total). Cervus reported that 54 % of the 842 paternity assignments had ≥95 % confidence, and the remaining 46 % of the paternity assignments had 80–94 % confidence. Eight percent of the seedlings resulted from selfing. We omitted from further analysis the 123 seedlings (13 %) that we were unable to successfully assign to a single father at ≥80 % confidence.

We quantified male reproductive success by estimating the total number of seeds sired by each of the 49 pollen donors on each day of the experiment. An unbiased estimate of siring success must incorporate the number of seeds successfully genotyped in each fruit as well as the total number of seeds from which that sample was drawn (Conner *et al.* 1996; Karron and Mitchell 2012). We genotyped 4–5 seeds per fruit and multiplied each donor’s proportion of siring by the number of seeds counted in that fruit. We then summed the estimated number of seeds sired by each donor across the 49 maternal plants to obtain total siring success for each donor on each day of the study. We assessed mate number per fruit by calculating the number of unique fathers (including self) that sired seeds in each fruit.

### Data analyses

We compared seed production, siring success, mate number and pollinator visitation between plants on the edge versus interior of the array using ANOVAs. We categorized the 24 plants around the perimeter of the square array as ‘edge’ plants, and the 25 plants in the centre of the array as ‘interior’ plants. For each response variable, we tested for an effect of spatial position in the array (edge vs. interior), the experimental day and an interaction between the two factors.

We compared gene dispersal distance and pollinator movement between plants on the edge versus the interior of the array. To do this, we calculated the distance between a seedling’s maternal and paternal parents. We used a *G*-test (likelihood ratio test) to evaluate whether the distributions of gene dispersal distances differed for edge and interior plants. We used a contingency test to examine whether pollinator flight segments differed between the edge and interior. To do this, we examined an individual bee’s foraging itinerary: every time it departed an edge plant, we calculated the distance it flew to the next plant visited. We did this for all bees that visited the array. We then performed the same procedure for departures from interior plants. We also calculated the distances of pollinator flight segments from the individual bee itineraries in the experimental array and also tested this with a contingency test. Analyses were performed in JMP, Version 14.2 (SAS Institute Inc., Cary, NC, 1989–2019) and R v.3.6.1 (R Core Team 2019).

## Results

### Female reproductive success

**Figure 2.**
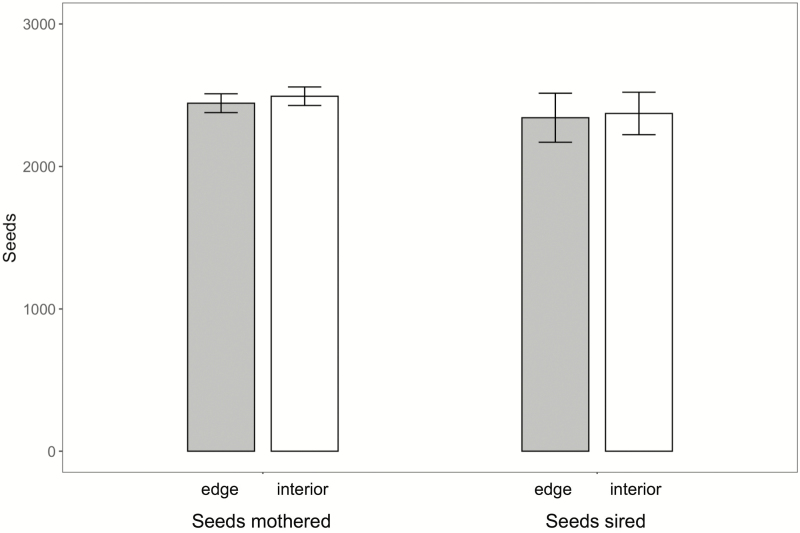
Mean number of seeds (±1 SE) mothered on, or sired by, edge and interior plants. *N* per bar = 96 for edge and 100 for interior plants. The number of seeds sired is slightly lower than the number of seeds mothered due to the estimation error of paternity shares.

We found no difference in the number of seeds per fruit mothered by edge and interior plants, although there were significant differences among days (Table 1; Fig. 2). The mean number of seeds per fruit mothered by edge plants was 2444 ± 66, versus 2493 ± 65 for interior plants, a difference of <2.0 %. Seed number declined steadily across the four sampling days, ranging from 2785 ± 93 (mean ± SE) on the first sampling day to 2087 ± 93 on the last sampling day. The lack of a significant interaction indicates that the rate of decline over time did not differ for edge and interior plants.

**Table 1.** ANOVA for effects of spatial location and day on the number of seeds mothered, the number of seeds sired and the number of pollen donors siring seeds within fruits. Significant (*P* < 0.05) results are highlighted in bold.

		Seeds mothered (error df =186)		Seeds sired (error df = 188)		Sires per fruit (error df = 179)	
Source	df	*F*	*P*	*F*	*P*	*F*	*P*
Edge or interior	1	0.24	0.62	0.06	0.81	0.48	0.49
Day	3	10.58	**<0.0001**	1.64	0.18	4.85	**0.003**
Interaction	3	1.73	0.16	0.95	0.42	1.70	0.17

The difference between seeds mothered by edge plants versus interior plants was <1 % of the total seed number. The small standard errors on these estimates confirm that our analyses were strong enough to detect even minor (3.5 %) effects of position on seeds mothered; the realized power of this analysis was sufficient to detect a true difference of 92 seeds.

### Male reproductive success

The number of seeds sired by individual plants did not differ between edge and interior plants (Table 1; Fig. 2). The mean number of seeds sired by edge plants was 2342 ± 172, versus 2372 ± 149 for interior plants, a difference of <2.2 %. Seeds sired did not vary among days (but showed a declining trend parallel to that for seeds mothered): the mean number of seeds sired on the first sampling day was 2604 ± 228, and on the last sampling day the average was 1975 ± 154. There was not a significant interaction of day and spatial location.

The difference between seeds sired by edge versus interior plants was <2.2 % of the total seed number, with small standard errors. The analysis was therefore strong enough to detect a moderate effect (8 %) of position on siring; the realized power of this analysis was enough to detect a true difference of 212 seeds.

### Number of mates siring seeds within fruits

**Figure 3.**
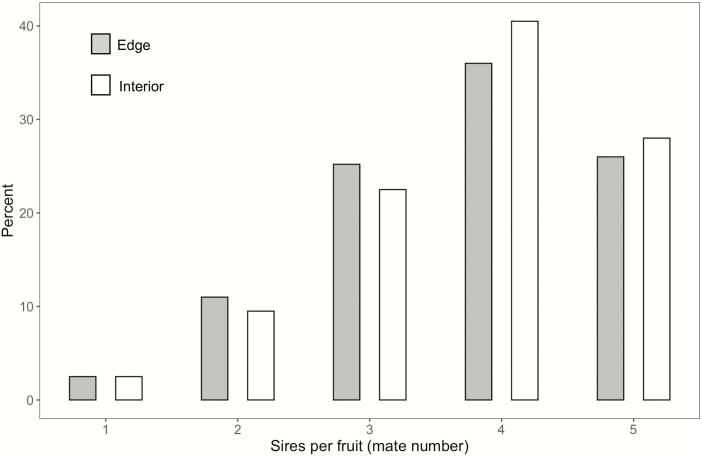
Mate number per fruit for edge versus interior plants. Mate number determined by paternity exclusion for 5 seeds per fruit. *N* = 92 plants for edge and 95 for interior.

Nearly all fruits were multiply sired (consisted of half sibs rather than full sibs) (Fig. 3). The number of pollen donors siring seeds within fruits did not differ significantly by spatial location, with the mean number of sires for edge plants being 3.72 ± 0.11, and that for interior plants being 3.83 ± 0.10, a difference of <2.9 % (Table 1; Fig. 3). For both spatial locations most fruits had 3–5 sires. The number of sires per fruit varied significantly among days with no discernable temporal trend, and there was no significant interaction of spatial location and day.

### Pollinator movements and pollen-mediated gene dispersal

During each day of our study flowers were open and receptive for ~4.5 h (from 530 to 1000 h local daylight savings time). The four 15-min pollinator observation periods each day represented ~22 % of all floral visits to our study plants. Over the 4 days we observed 209 floral visits: 88 % by *B. vagans* workers, 10 % by *Bombus impatiens* workers and 2 % by unidentified *Bombus* workers. The mean rate of pollinator visitation to flowers on edge plants was lower than to flowers on interior plants: edge, 1.30 ± 0.11 visits per flower per hour; interior, 1.53 ± 1.20 visits per flower per hour, a difference of >15 %.

**Figure 4.**
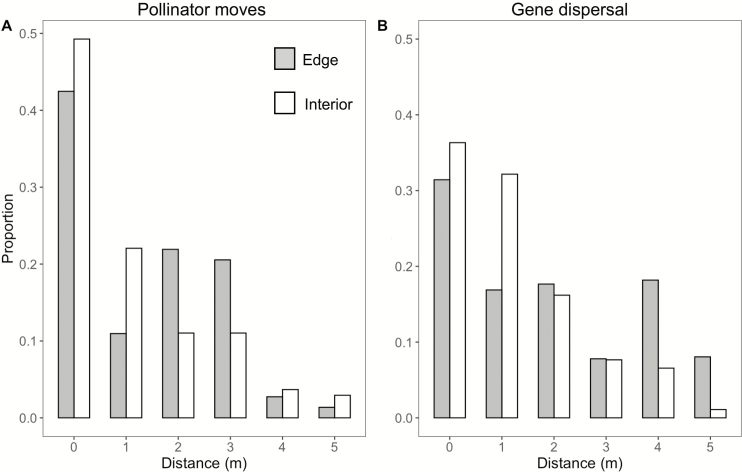
Pollinator movements and pollen-mediated gene dispersal for edge and interior plants. X-axis categories are abbreviated; true ranges are 0–0.999, 1–1.999, etc. *N* for pollinator moves = 73 for edge plants and 136 for interior. *N* for gene dispersal = 385 for the edge and 457 for the interior.

Both pollinator flight segments and pollen-mediated gene dispersal were highly restricted for both edge and interior plants (Fig. 4). Edge and interior plants differed significantly in the pattern of pollinator moves (likelihood ratio χ ^2^ = 71.5, 5 df, *P* < 0.0001). This mostly reflects longer moves for edge plants: 54 % of pollinator flights departing edge plants were <2 m away, compared to 71 % for flights departing interior plants.

The pattern of pollen-mediated gene dispersal also varied significantly between edge and interior plants (Fig. 4 a,b, likelihood ratio χ ^2^ = 11.1, 5 df, *P* < 0.049). This reflects more extensive gene dispersal for edge plants; the mean distance of pollen-mediated gene dispersal from flowers on edge plants was 2.38 ± 0.08 m, whereas the mean distance of pollen-mediated gene dispersal from flowers on interior plants was 1.70 ± 0.06 m. For both pollinator moves and gene dispersal, edge plants deviated more from a smooth decline with distance, showing a distinct increase in moves and dispersal >2 m (Fig. 4 a,b) relative to interior plants.

## Discussion

Although edge effects are widely assumed to influence plant–pollinator interactions and plant reproductive success, we found no significant differences between edge and interior plants in the number of seeds sired, the number of seeds mothered or the number of pollen donors siring seeds within fruits. However, there were subtle effects of plant spatial location on pollinator foraging behaviour and on the distance of pollen-mediated gene dispersal.

Edge effects on plant reproductive success should be most pronounced in species with limited pollen carryover. Since pollen carryover is extremely limited in *M. ringens* (Holmquist *et al.* 2012; Mitchell *et al.* 2013), our study system was well-suited for detection of edge effects on plant reproductive success. Thus, the lack of edge effects on male and female reproductive success in an especially susceptible situation suggests that spatial location within a population will not necessarily influence these aspects of plant reproduction.

Our findings are in contrast to those of Ison and Wagenius (2014), who found that seed set in self-incompatible *Echinacea angustifolia* was significantly lower for plants on the edge versus the interior of an experimental plot. They noted a significant interaction between plant location and flowering date, which they attributed to increased pollen limitation later in the flowering season. By contrast, we found no effect of plant spatial location on seed set and no evidence for pollen limitation (see below). Therefore, edge effects may appear only under certain conditions, such as pollen limitation.

One noticeable effect of the edge position in our study concerned pollen-mediated gene dispersal, for which we found meaningful differences between edge and interior plants, with edge plants showing more idiosyncratic patterns and an increase in the weight of the tails of the distribution. This finding should inspire caution in studies of gene dispersal and warrants more attention to potential position effects. This applies to both field studies and array experiments. As noted by Levin (1995), edge plants may play an important role in pollen-mediated gene dispersal. Occasional long-distance pollen-mediated gene dispersal from edge plants could reduce the extent of fine spatial population genetic structure. This may be particularly true in populations with a high proportion of edge plants, such as long linear populations.

We found that pollinator visitation rate was lower for edge than for interior plants. This result contrasts with the intuition that edge plants might have higher visitation because they are the first plants that a visitor arriving from elsewhere would encounter (Levin 1995). Therefore, edge plants may not act as a ‘buffer’ or barrier that captures any interpopulation pollen movement. It also suggests that pollinators may aggressively restrict movements between populations and avoid population edges. In fact, in patchily distributed populations, bees do not forage in a linear fashion, but rather turn back to the patch interior to avoid the cost of travelling longer distances between patches, resulting in decreased visitation to the edge (Rasmussen and Broedsgaard 1992), although these behaviours are species-specific (Brunet *et al.* 2019).

Although we found fairly strong effects of plant spatial location on pollinator behaviour, this did not translate into strong effects on seed production and mating patterns, with the exception of gene dispersal. These results are similar to Kunin (1997), who found higher pollinator constancy in the interior of a *Brassica kaber* experimental plot, but no differences in visitation rate or seed set. Our findings highlight the value of a holistic investigation of pollination, since the potential for effects on reproductive success may differ from effects on mate composition and pollen-mediated gene dispersal (Page *et al.* 2019). Our findings also provide insight on the mechanistic connections between these responses. Although pollinator visitation was lower for edge plants, it was still relatively high, such that edge flowers received on average over five visits (1.3 visits per flower per hour over 4.5 h), which is more than enough to saturate the pollen dose–response relationship (Karron *et al.* 2006). Thus, edge plants were not pollen-limited, so seed production was not lower than for interior plants (which received nearly seven visits). However, this raises the possibility that interior plants might have more scope for mate choice (since a smaller fraction of pollen on stigmas could be successful in fertilization; Christopher *et al.* 2019). Our data do not allow us to test this possibility.

It is possible that the spacing between plants or population size might affect our conclusions. It would be informative to compare pollinator behaviour, seed production, and mate diversity for edge versus interior plants in a variety of population densities and sizes in our system. For example, increased density or the presence of a co-flowering species can alter the patterns of pollinator visitation, constancy and seed set (Kunin 1997; Thomson *et al.* 2019). Aggregated plant distributions can also decrease pollinator flight distances (Cresswell 2000), which may have implications for pollinator-mediated gene dispersal.

Because plant populations are finite and often irregularly shaped, a large fraction of wild plants will be on or near edges. This is especially true for linear populations (e.g. along stream courses or hedgerows). Plants on the edge of experimental populations are widely assumed to experience different pollination environments than those in the interior because of edge effects (Fagan *et al.* 1999; Ricketts 2001). If true, this would complicate interpretation of many studies. Furthermore, since natural populations differ greatly in population shape, size and density, context specificity of conclusions would be likely and require consideration. However, our results suggest that, despite the conservative intuition of many researchers, edge effects in plant reproduction may be minor, and restricted only to some aspects of pollination biology.

Our findings also have important implications for the design of experimental arrays, which are increasingly used for studies of natural selection (Caruso *et al.* 2019). Such studies of necessity assume that all plants are equal except concerning the traits under study, and therefore assume that there are no edge effects. Large edge effects would muddy estimates of selection and complicate interpretation of results. Our findings suggest that edge effects will not necessarily play a large role in experimental arrays.

## Conclusion

We found no differences in fitness between edge and interior plants in our experimental array. We did find lower pollinator visitation rates to edge plants, and the shape of the pollen-mediated gene dispersal curve differed between edge and interior plants. Taken together, these results suggest that edge effects are not as strong or ubiquitous as commonly assumed, and that different plant reproduction parameters respond to spatial locations independently (Ries *et al.* 2017).

## Data Archiving

Data are available on dryad, doi:10.5061/dryad.9s4mw6mcx.
